# Clinical Characteristics and Treatment Courses of Exceptional Long-Term Survivors After Multimodal Therapy Including Proton Beam Therapy for Borderline Resectable and Locally Advanced Pancreatic Cancer: A Retrospective Case Series

**DOI:** 10.7759/cureus.115391

**Published:** 2026-08-29

**Authors:** Haruko Numajiri, Masashi Mizumoto, Osamu Shimomura, Yuya Hagiwara, Masahiko Harada, Hikaru Niitsu, Taisuke Sumiya, Masaaki Goto, Takuya Sawada, Takeshi Fujisawa, Keiichiro Baba, Masatoshi Nakamura, Takashi Saito, Kei Nakai, Hideyuki Sakurai

**Affiliations:** 1 Department of Radiation Oncology, University of Tsukuba Hospital, Tsukuba, JPN; 2 Department of Gastrointestinal-Hepato-Biliary-Pancreatic Surgery, University of Tsukuba Hospital, Tsukuba, JPN; 3 Department of Gastroenterology, University of Tsukuba Hospital, Tsukuba, JPN

**Keywords:** concurrent chemoradiotherapy, conversion surgery, locally advanced pancreatic cancer, multidisciplinary treatment, pancreatic cancer, proton beam therapy

## Abstract

Background

Despite advances in multimodal treatment, the prognosis of borderline resectable pancreatic cancer (BR-PC) and unresectable locally advanced pancreatic cancer (UR-LAPC) remains poor. We performed a descriptive retrospective analysis of 10 exceptional long-term survivors who survived more than five years following proton beam therapy (PBT).

Methods

Among 112 patients with BR-PC or UR-LAPC who underwent definitive PBT at our institution, 10 patients who survived for more than five years were retrospectively analyzed. Clinical characteristics, treatment courses, adverse events, and outcomes were reviewed.

Results

The median age was 64.5 years (range, 50-74), and two patients were men and eight were women. Six patients had BR-PC and four had UR-LAPC. The median pretreatment CA19-9 level was 78.2 U/mL (range, <2.0-837). Three patients received induction chemotherapy for less than one month. All patients received PBT at 67.5 Gy (RBE) in 25 fractions with concurrent gemcitabine and hyperthermia, followed by maintenance chemotherapy. The median observed follow-up/survival duration was 76 months (range, 60-115 months). Three patients underwent conversion surgery following PBT, with pathological complete response achieved in two patients. Among the seven patients who did not undergo surgery, five experienced disease progression, whereas two remained progression-free throughout follow-up. At the time of analysis, eight of the 10 patients were alive.

Conclusions

Long-term survival beyond five years was observed in a subset of patients with BR-PC or UR-LAPC treated with multimodal therapy including PBT. Clinical courses varied substantially among these exceptional survivors, including both patients who underwent conversion surgery and those managed without surgical resection. Further studies are needed to clarify factors associated with long-term survival.

## Introduction

Pancreatic cancer remains one of the most aggressive malignancies and is associated with an extremely poor prognosis despite recent advances in multidisciplinary treatment [1,2]. Surgical resection offers the only potentially curative treatment; however, approximately half of patients present with metastatic disease at diagnosis, while a substantial proportion have borderline resectable (BR-PC) or unresectable locally advanced pancreatic cancer (UR-LAPC), precluding upfront surgery [3].

Recent advances in systemic chemotherapy, including FOLFIRINOX and gemcitabine plus nab-paclitaxel, have substantially improved the treatment of pancreatic cancer and have become the standard regimens for patients with BR-PC and UR-LAPC [4,5]. In addition, multidisciplinary treatment strategies combining chemotherapy, radiotherapy, and surgery have increased the feasibility of conversion surgery in selected patients, contributing to prolonged survival [6-8].

Radiotherapy has become an important component of local treatment for pancreatic cancer [9,10]. Among available modalities, proton beam therapy (PBT) enables delivery of high radiation doses while reducing radiation exposure to surrounding gastrointestinal organs, thereby potentially improving local tumor control with acceptable toxicity [11]. PBT has shown promising clinical outcomes in the treatment of pancreatic cancer [12]. However, little is known about the clinical characteristics and treatment courses of patients who achieve exceptionally long-term survival following PBT.

Because long-term survival in BR-PC and UR-LAPC is exceptionally rare, characterization of exceptional survivors may provide descriptive insights into the diverse clinical courses and treatment patterns observed among patients who survive beyond five years after PBT. Therefore, we retrospectively investigated the clinical characteristics, treatment courses, and outcomes of patients who survived for more than five years following PBT as part of multidisciplinary treatment.

## Materials and methods

Patient selection

We retrospectively reviewed 112 consecutive patients with BR-PC or UR-LAPC who underwent PBT at the University of Tsukuba Hospital between April 2013 and May 2021. Among them, patients who achieved long-term survival (>5 years after PBT) were selected for inclusion in this retrospective case series.

The diagnosis of pancreatic cancer was based on histopathological confirmation whenever available. In cases without definitive pathological confirmation, pancreatic cancer was diagnosed clinically based on comprehensive assessment, including imaging findings showing a hypovascular pancreatic mass with invasive features (such as extrapancreatic extension and/or vascular involvement), elevated tumor-associated biomarkers (including CA19-9 and/or elastase-1), and the overall clinical course. Such cases were reviewed through multidisciplinary evaluation before inclusion in the study.

Resectability status was determined retrospectively based on the NCCN Clinical Practice Guidelines in Oncology: Pancreatic Adenocarcinoma (Version 2.2026, April 22, 2026) [13].

Although treatment decisions at the time were made through multidisciplinary oncology conferences according to the standards available during each period, all cases included in the present analysis were re-evaluated using a uniform set of criteria based on review of clinical records and conference documentation.

Clinical information was obtained from electronic medical records and institutional databases. Treatment strategies and follow-up plans were determined through multidisciplinary discussion according to institutional practice during the study period.

Treatment and follow-up

Proton beam therapy was delivered according to the protocol of our institution, including target delineation, treatment planning, and respiratory motion management procedures. Details of the PBT technique and concurrent chemotherapy regimen have been described previously [14]. All prescribed proton doses are expressed as Gy(RBE), assuming a standard proton relative biological effectiveness (RBE) of 1.1.

Following PBT, maintenance chemotherapy was administered according to prevailing standards of care during the study period. Regimens included gemcitabine monotherapy (GEM), gemcitabine plus S-1 (GS), and gemcitabine plus nab-paclitaxel (GnP), based on established evidence for unresectable pancreatic cancer [5,15-17].

After completion of PBT, patients were generally followed with serum tumor marker measurements every 1-2 months and contrast-enhanced CT examinations every 3-4 months. Tumor status was evaluated primarily by contrast-enhanced CT, supplemented by additional imaging studies when clinically indicated. Disease progression was defined according to RECIST version 1.1 criteria [18]. Elevation of tumor markers alone was not considered evidence of disease progression unless accompanied by radiological findings consistent with progressive disease.

Treatment-related adverse events were retrospectively evaluated based on medical records and graded according to the Common Terminology Criteria for Adverse Events (CTCAE) version 5.0 [19]. Adverse events occurring within three months after completion of proton beam therapy were defined as acute adverse events, whereas those occurring more than three months after completion of proton beam therapy were defined as late adverse events.

Data collection

The following clinical data were collected: age, sex, tumor location, disease stage, serum CA19-9 level, chemotherapy regimen, PBT dose, conversion surgery, pathological findings, disease progression, subsequent treatments, survival outcomes, and treatment-related toxicities. Individual treatment courses were also reviewed to characterize the clinical features of long-term survivors.

Outcomes

The primary objective of this study was to describe the clinical characteristics and treatment courses of patients who survived for more than five years after PBT combined with chemotherapy. The secondary objective was to evaluate outcomes, including conversion surgery, patterns of disease progression, subsequent treatments, survival status, and treatment-related toxicities.

Because of the descriptive nature of this study and the limited sample size, no formal statistical analyses were performed.

Ethics

All patients had provided written comprehensive consent for registration in the institutional clinical database and for the use of their clinical information, imaging findings, and treatment course for research and academic publication.

## Results

Patient characteristics

Ten patients who survived for more than five years after PBT combined with chemotherapy were identified during the study period. Patient characteristics are summarized in Table 1.

**Table 1:** Patient characteristics. SMA, superior mesenteric artery; CA, celiac axis; CHA, common hepatic artery; PV, portal vein; SMV, superior mesenteric vein; PV-SMV confluence, portal vein-superior mesenteric vein confluence; BR, borderline resectable; UR, unresectable;  EUS-FNA, endoscopic ultrasound-guided fine-needle aspiration; CA19-9, carbohydrate antigen 19-9. *Unresectable celiac axis involvement was confirmed during exploratory laparotomy.

Case	Age (years)	Sex	ECOG PS	tumor location	tumor size (mm)	Arterial involvement	Venous involvement	NCCN BR/UR status	Clinical TNM stage	Diagnostic pathology	CA19-9 (U/mL)
1	52	F	0	Body	41	SMA≤180	PV, SMV	BR	cT3N0M0	EUS-FNA: Adenocarcinoma	114.6
2	61	F	0	Head	30	SMA>180	SMV	UR	cT4N1M0	EUS-FNA: Adenocarcinoma	22.5
3	70	M	0	Body	24	CA≤180, CHA involvement (reconstructable)	PV-SMV confluence	BR	cT3N0M0	Pancreatic juice cytology: Class III	146.5
4	74	M	0	Head	22	SMA≤180, CA≤180	PV-SMV confluence	BR	cT3N0M0	EUS-FNA: Non-diagnostic	40.5
5	64	F	0	Head	38	SMA>180, CA>180		UR	cT4N0M0	EUS-FNA: Adenocarcinoma	246
6	58	F	0	Body	30	SMA≤180, CA≤180	PV	BR	cT3N0M0	EUS-FNA: Class III	15.8
7	67	F	0	Head	23	CHA involvement (unreconstructable)	PV	UR	cT4N0M0	EUS-FNA: Adenocarcinoma	41.8
8	65	F	0	Body	20	CA involvement*		UR	cT4N1M0	Exploratory laparotomy biopsy: Adenocarcinoma	<2.0
9	70	F	0	Head	18	CHA involvement (reconstructable)	PV	BR	cT3N0M0	EUS-FNA: Adenocarcinoma	281
10	50	F	0	Head	26	CHA involvement (reconstructable)	SMV	BR	cT3N0M0	EUS-FNA: Adenocarcinoma	837

Among these patients, there were two men and eight women, with a median age of 64.5 years (range, 50-74 years). Six patients had borderline resectable pancreatic cancer (BR-PC), and four had unresectable locally advanced pancreatic cancer (UR-LAPC) according to the NCCN Guidelines (Version 2.2026). The median pretreatment serum CA19-9 level was 78.2 U/mL (range, <2.0-837 U/mL). Histological confirmation of adenocarcinoma was obtained in seven patients by endoscopic ultrasound-guided fine-needle aspiration (EUS-FNA). In one additional patient, EUS-FNA was non-diagnostic because a cystic lesion located between the duodenum and the tumor prevented adequate tissue sampling; however, adenocarcinoma was subsequently confirmed by biopsy of a cutaneous metastasis that developed after treatment. One patient had Class III cytology on EUS-FNA, and another had Class III cytology on pancreatic juice cytology. In these three patients without a definitive pathological diagnosis at the time of initial presentation, pancreatic cancer was diagnosed based on the overall assessment of clinical findings, radiological features, and tumor marker levels. 

Treatment courses

The treatment courses of all patients, including chemotherapy regimens, PBT dose, treatment-related toxicities, and subsequent clinical outcomes, are summarized in Table 2 and illustrated in Figure 1.

**Table 2:** Treatment details and clinical outcomes. PBT, proton beam therapy; GS, gemcitabine plus S-1; GnP, gemcitabine plus nab-paclitaxel; GEM, gemcitabine; DP-PVR, distal pancreatectomy with portal vein resection and reconstruction; SSPPD, subtotal stomach-preserving pancreaticoduodenectomy; PD, pancreaticoduodenectomy. *PBT dose is presented as total prescribed dose/fractionation schedule. For example, 67.5 Gy/25 Fr indicates 67.5 Gy(RBE) delivered in 25 fractions. **Duration from PBT was calculated from initiation of PBT and rounded to the nearest month. All included patients survived for more than 5 years after PBT.

Case	Induction chemotherapy before PBT	Concurrent chemotherapy	Post-PBT Chemotherapy	PBT dose (Gy (RBE))^*^	Conversion surgery	Grade ≥3 adverse events	Time to progression (months)	Site of first progression	Subsequent treatment after progression	Status at last follow-up	Duration from PBT (months)^ **^
1	None	GEM	GnP	67.5Gy/25Fr	None	None	No progression at last follow-up	None	N/A (no progression)	Alive without disease	115
2	None	GEM	GnP	67.5Gy/25Fr	None	Vertebral compression fracture	39	Lung	Declined further anticancer treatment	Dead of pancreatic cancer	68
3	None	GEM	GS	67.5Gy/25Fr	None	None	26	Lung	S1	Alive with disease	103
4	None	GEM	GS	67.5Gy/25Fr	None	None	51	Lung	Declined further anticancer treatment	Dead of pancreatic cancer	84
5	None	GEM	GEM	67.5Gy/25Fr	None	None	50	Lung	GnP	Alive with disease	81
6	GS (2wk)	GEM	GnP	67.5Gy/25Fr	DP-PVR	Gastric bleeding	No progression at last follow-up	None	N/A (no progression)	Alive without disease	82
7	GnP (3wk)	GEM	GnP	67.5Gy/25Fr	None	None	No progression at last follow-up	None	N/A (no progression)	Alive without disease	71
8	None	GEM	GnP	67.5Gy/25Fr	None	None	37	Lung+Liver+LN	Clinical trial (outside institution)	Alive with disease	61
9	GnP (2wk)	GEM	GnP	67.5Gy/25Fr	SSPPD	None	No progression at last follow-up	None	N/A (no progression)	Alive without disease	63
10	None	GEM	GEM	67.5Gy/25Fr	PD	None	No progression at last follow-up	None	N/A (no progression)	Alive without disease	60

**Figure 1:**
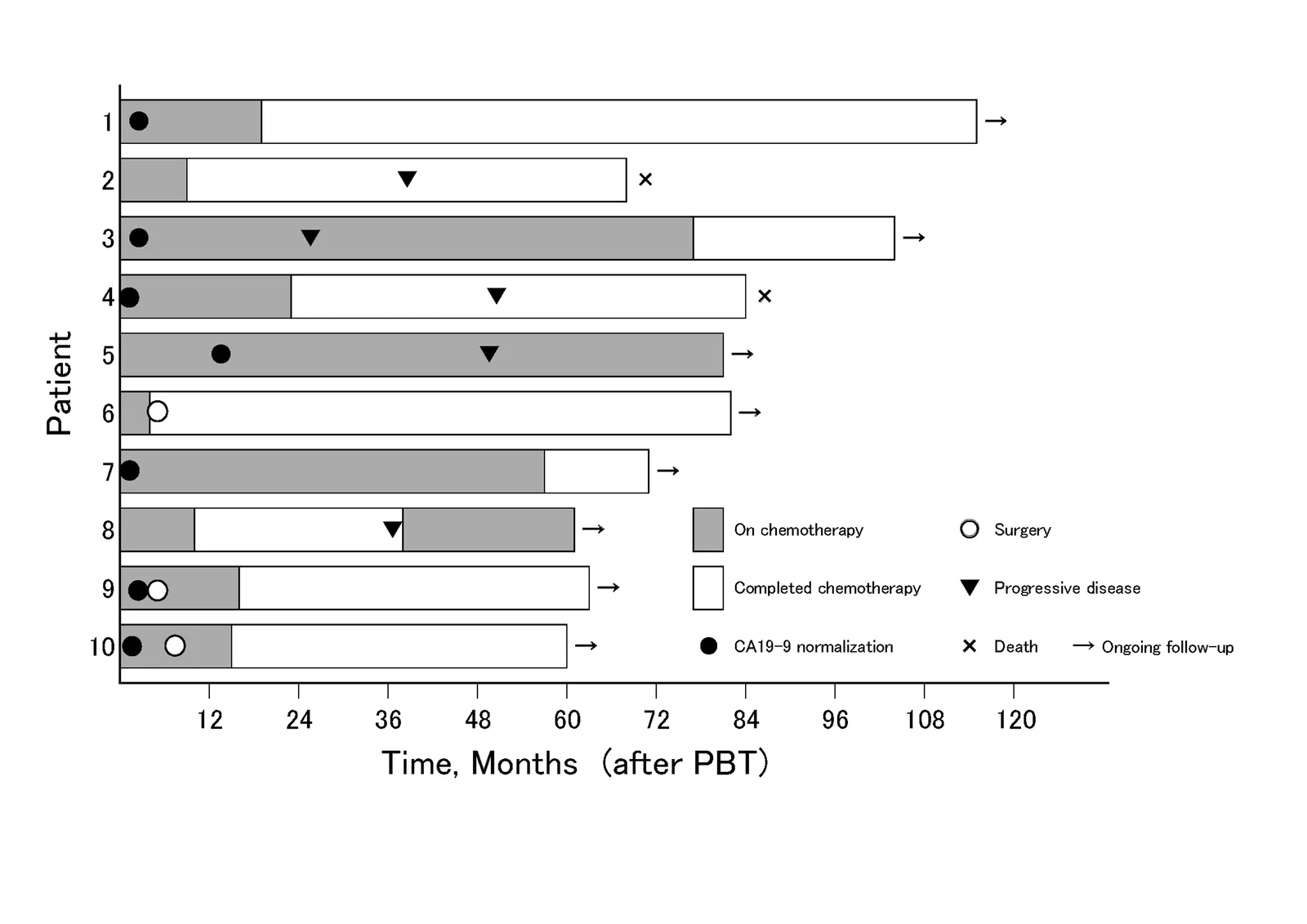
Swimmer plot illustrating the clinical courses of the ten long-term survivors after proton beam therapy (PBT) combined with chemotherapy. Gray bars indicate periods of chemotherapy, and white bars indicate follow-up after completion of chemotherapy; symbols denote carbohydrate antigen 19-9 (CA19-9) normalization (●), conversion surgery (○), disease progression (▼), death (×), and ongoing follow-up (→). Cases 2, 6, and 8 had normal CA19-9 levels at diagnosis. Therefore, no CA19-9 normalization date is indicated.

Three patients received induction chemotherapy for less than one month. All patients received PBT at 67.5 Gy (RBE) in 25 fractions with concurrent gemcitabine and regional hyperthermia, followed by maintenance chemotherapy.

The median follow-up duration was 76 months (range, 60-115 months). At the time of analysis, eight of the ten patients were alive. The clinical course and imaging findings of the representative case (Case 1) are shown in Figure 2.

**Figure 2:**
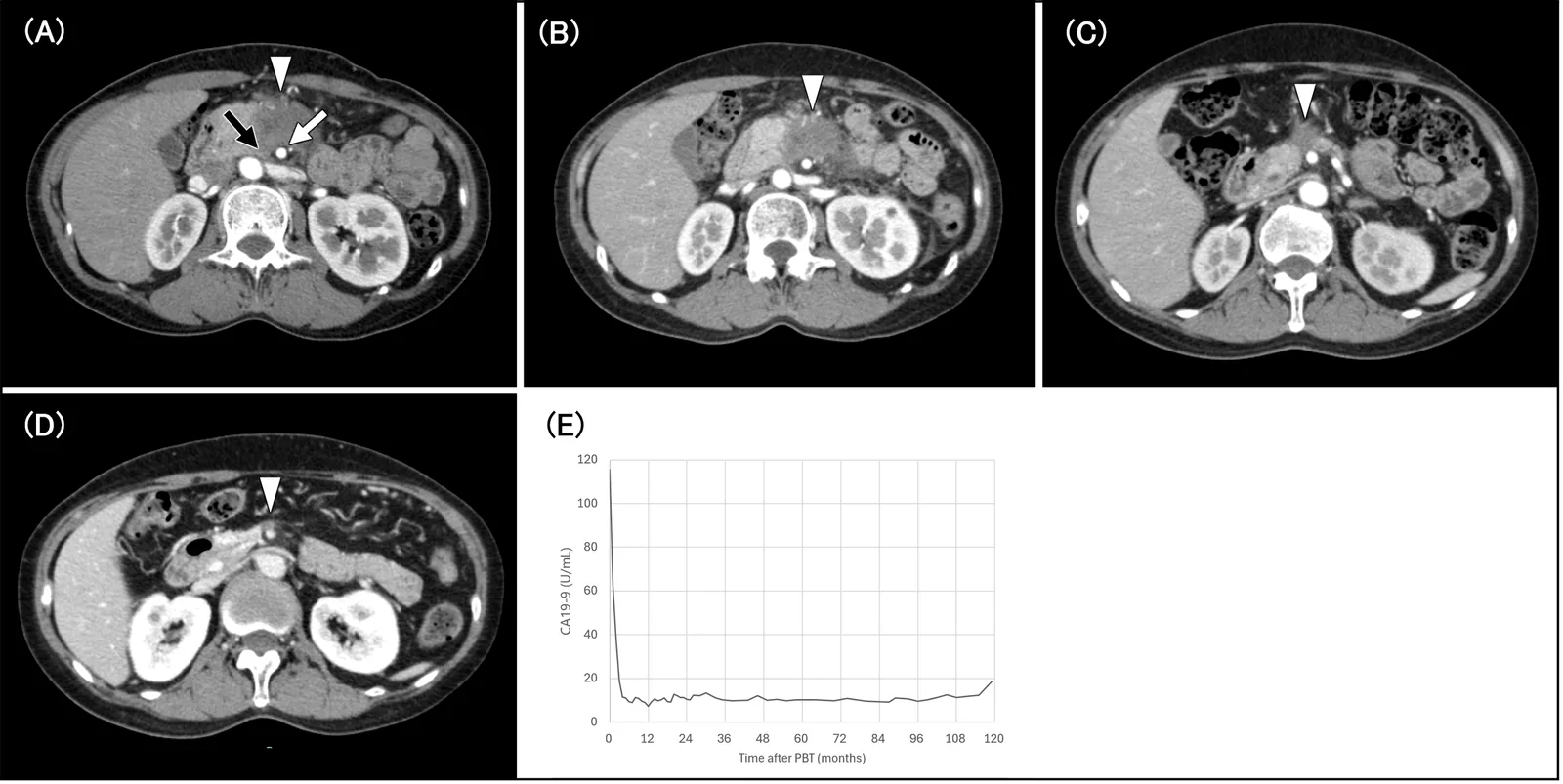
Representative case demonstrating durable local control after proton beam therapy (PBT). (A) Pretreatment contrast-enhanced CT showing a hypovascular pancreatic body tumor (arrowhead) with obliteration of the superior mesenteric vein (black arrow) and abutment of the superior mesenteric artery (white arrow). (B) CT obtained 2 weeks after completion of proton beam therapy (PBT) showing slight tumor shrinkage (arrowhead). (C) CT obtained 19 months after PBT, at completion of chemotherapy. Although a small residual soft-tissue lesion (arrowhead) persisted, no disease progression was observed. (D) CT obtained 115 months after PBT demonstrating durable local control (arrowhead) without disease progression. (E) Serum carbohydrate antigen 19-9 (CA19-9) levels during treatment and follow-up. The level progressively decreased during gemcitabine plus nab-paclitaxel therapy and normalized 3 months after PBT.

Post-PBT clinical course

Three of ten patients underwent conversion surgery after PBT. During the study period, no predefined institutional criteria for conversion surgery had been established. Surgical decisions were made on a case-by-case basis through multidisciplinary discussion, and patients showing favorable radiographic response and deemed technically resectable were considered surgical candidates. Detailed information regarding the surgical patients, including the surgical procedure, interval from PBT initiation to surgery, pathological response (Evans grade), ypTNM stage, margin status, nodal findings, postoperative complications, and postoperative systemic therapy, is summarized in Table 3. One patient who underwent portal vein reconstruction developed postoperative gastric remnant ischemia. No major postoperative complications occurred in the remaining patients. Notably, the same patient subsequently experienced recurrent hyperammonemia requiring hospitalization beginning 5.7 years after surgery. Postoperative systemic therapy was administered in two of the three surgical patients.

**Table 3:** Detailed information of surgical patients. DP-PVR, distal pancreatectomy with portal vein resection and reconstruction; SSPPD, subtotal stomach-preserving pancreaticoduodenectomy; PD, pancreaticoduodenectomy. All surgical and circumferential resection margins were negative.

Case	Surgical procedure	Time from PBT initiation to surgery (months)	Pathological response (Evans grade)	ypTNM	margin status	Positive/examined nodes	postoperative complications	postoperative systemic therapy
6	DP-PVR	5	III (carcinoma in situ only)	ypTisN0M0	R0	0/9	Gastric remnant ischemia (Clavien-Dindo grade IIIa)	None
9	SSPPD	5	III	ypT1aN0M0	R0	0/20	None	S1
10	PD	8	IV	ypT0N0M0	R0	0/7	None	S1

Five of ten patients experienced disease progression during follow-up. Subsequent management consisted of S-1 chemotherapy in one patient, gemcitabine plus nab-paclitaxel in one patient, enrollment in a clinical trial at another institution in one patient, whereas two patients declined further anticancer treatment after progression. Detailed treatment timelines are presented in the swimmer plot.

Treatment-related toxicity

Treatment-related toxicities were generally mild. One patient developed Grade 3 gastric antral bleeding during follow-up after conversion surgery with portal vein resection and reconstruction. Given the patient's surgical history, the bleeding was not necessarily attributable solely to PBT, and post-surgical changes in portal venous circulation may have been an additional contributing factor. In addition, one patient experienced a Grade 3 vertebral compression fracture within the irradiated field that required hospitalization. However, multiple additional vertebral fractures subsequently occurred outside the irradiated field, suggesting that underlying osteoporosis may also have contributed to the development of these fractures. No other Grade 3 or higher toxicities potentially related to PBT were observed.

## Discussion

The present study describes the clinical characteristics and treatment courses of ten patients who survived for more than five years following PBT combined with chemotherapy for pancreatic cancer. Among the long-term survivors in the present series, three underwent conversion surgery, two of whom achieved pathological complete response. Importantly, long-term survival was observed not only in patients who underwent conversion surgery but also in selected patients managed without surgical resection. This observation is consistent with previous reports demonstrating that durable survival can be achieved in selected patients with locally advanced pancreatic cancer treated with PBT combined with chemotherapy [14].

Conversion surgery has emerged as an important treatment strategy for selected patients with borderline resectable or locally advanced pancreatic cancer who respond favorably to multidisciplinary treatment [6,7,20]. Previous studies from our institution have demonstrated that surgical resection after high-dose PBT can be performed safely and may result in substantial pathological tumor regression, including pathological complete response in selected patients [21]. However, one of the patients who achieved pathological complete response in the present series lacked definitive histological confirmation of adenocarcinoma before treatment. Although the resected specimen demonstrated only carcinoma in situ without residual invasive carcinoma, the interpretation of this case as a treatment-induced pathological complete response should be made with caution because of the absence of unequivocal pretreatment pathological confirmation. Although several patients overlapped with the cohort reported by Shimomura et al. [21], that study focused on the safety and pathological outcomes of resection after high-dose PBT, whereas the present study specifically examined long-term survivors exceeding five years after PBT. Taken together, these findings are consistent with previous reports describing conversion surgery among selected patients who achieved long-term survival following multidisciplinary treatment [6].

Interestingly, two patients who did not undergo surgical resection remained free from disease progression for more than five years after PBT. Persistent soft-tissue attenuation around major vessels was observed on follow-up imaging despite prolonged disease control. These observations suggest that persistent radiographic abnormalities following PBT do not necessarily represent residual viable tumor and underscore the challenges of interpreting post-treatment imaging after high-dose radiotherapy, which is consistent with previous reports describing the difficulty of differentiating treatment-related fibrosis from viable tumor after neoadjuvant therapy and radiotherapy [22,23]. Despite these notable imaging findings, no consistent factors associated with long-term survival without surgery were identified. In particular, there were no clear shared clinical characteristics, including baseline radiological findings, tumor marker levels, or duration of systemic therapy. Although both patients developed Grade 2 duodenal ulcers after PBT, the significance of this observation remains uncertain because identical dose constraints were applied throughout the cohort.

Therefore, the factors that enable long-term survival without surgery remain unclear. Further analyses comparing these exceptional survivors with the overall cohort will be required to better understand factors associated with long-term survival, including tumor biology, treatment patterns, and patient selection.

Five of ten patients experienced disease progression during follow-up but nevertheless achieved prolonged survival. Post-progression management varied substantially among patients and included additional chemotherapy, participation in a clinical trial, or observation without further anticancer treatment. Therefore, the mechanisms underlying prolonged survival after progression remain unclear.

Notably, all long-term survivors in the present series received little or no induction chemotherapy before PBT. However, this observation should be interpreted cautiously. Patients considered to be at higher risk of occult metastatic disease were more likely to receive induction systemic chemotherapy in clinical practice, potentially introducing selection bias. Furthermore, because survival was calculated from the initiation of PBT rather than from the time of diagnosis, prolonged survival among patients receiving induction chemotherapy may not be fully captured. The study period also extended over a decade, during which treatment strategies for pancreatic cancer evolved substantially. The absence of induction chemotherapy among the long-term survivors in the present series may partly reflect historical treatment practices rather than a biological or clinical characteristic required for long-term survival. Therefore, our findings should not be interpreted as evidence that omission of induction chemotherapy improves outcomes. Future analyses of the entire treated cohort may help elucidate the impact of treatment era and treatment sequencing on long-term outcomes.

Among these selected long-term survivors, treatment-related toxicity was generally manageable. However, because the present study included only patients who survived longer than five years, the findings should not be interpreted as representative of the overall safety profile of PBT. Assessment of treatment-related toxicity in the entire treated cohort would be required to adequately evaluate treatment safety.

Because the primary objective of this study was to characterize exceptional long-term survivors rather than to evaluate survival outcomes in the entire treated population, analyses of overall survival and 5-year survival rates were not performed. In addition, complete survival follow-up information was not available for all patients in the source cohort. Therefore, a formal survival analysis of the entire cohort was beyond the scope of the present study. Further investigation of the full cohort may help clarify the incidence and predictors of long-term survival. Accordingly, the findings should be interpreted as descriptive observations of exceptional survivors rather than estimates of treatment efficacy.

Several patients included in the present study have been reported in previous institutional publications evaluating proton beam therapy and conversion surgery. However, those studies focused on treatment feasibility, safety, pathological response, and survival outcomes in broader treatment cohorts. In contrast, the present case series specifically investigates the clinical characteristics and treatment courses of patients who achieved survival beyond five years, providing insight into patterns of exceptional long-term survival that were not addressed in the previous reports.

This study has several limitations. First, it was a retrospective, single-institution case series with a limited number of patients, and no comparative analyses were performed. Given the small sample size and the descriptive nature of this retrospective case series, formal statistical analyses were not performed, and the findings should be considered hypothesis-generating rather than definitive. Second, because only long-term survivors were included, the findings should not be interpreted as evidence of treatment efficacy or superiority of PBT. In addition, the absence of a control group prevents any assessment of the relative efficacy of PBT compared with other treatment modalities. Therefore, the favorable outcomes observed in this study cannot be attributed solely to PBT and may have been influenced by patient selection, tumor biology, chemotherapy, conversion surgery, and other unmeasured factors. Third, one patient who achieved pathological complete response lacked definitive pretreatment histological confirmation of adenocarcinoma. Therefore, the pathological response observed in this case should be interpreted with caution, although the resected specimen demonstrated carcinoma in situ without residual invasive carcinoma. In addition, decisions regarding conversion surgery were made on a case-by-case basis during the study period, and a reproducible selection algorithm cannot be retrospectively established. Furthermore, detailed information regarding all subsequent treatment modifications after disease progression was not uniformly available because of the retrospective nature of the study. Nevertheless, characterization of these exceptional long-term survivors illustrates the heterogeneous clinical courses observed among patients who survived longer than five years after PBT combined with chemotherapy, both with and without conversion surgery.

## Conclusions

Long-term survival beyond five years was observed in a small subset of patients with borderline resectable or locally advanced pancreatic cancer who received PBT combined with chemotherapy. Among these exceptional survivors, diverse clinical courses were observed, including both patients who underwent conversion surgery and those who achieved prolonged survival without surgical resection. Characterization of these long-term survivors may provide insights into the potential role of PBT within multidisciplinary treatment strategies for pancreatic cancer. Given the retrospective design and limited sample size of this study, further prospective studies with larger cohorts are warranted to clarify factors associated with long-term survival and to better define the role of PBT in this setting.
